# Supplementary material for: Testing structural identifiability by a simple scaling method
Source: PLoS Comput Biol. 2020 Nov 3;16(11):e1008248. doi: 10.1371/journal.pcbi.1008248 (PMC7665633; doi:10.1371/journal.pcbi.1008248)
Supplement: Attachment — Submitted filename: reply.pdf [file pcbi.1008248.s002.pdf]

Dear Editor of PLoS Computational Biology,

Thank you very much for handling the revision of our submitted manuscript.

We thank the reviewers for their thorough assessment of our work. Their comments have raised important questions and, in answering them, we believe that our work has improved substantially.

Here we summarize the changes made:

- We have rewritten some parts of the Abstract and Introduction to clarify the scope and main messages of our work. We have also devoted more space to define more rigorously concepts such as functional independence, elasticity or structural identifiability.
- We have re-organized the Main text to make it more self-contained. In particular, we have rewritten many parts of the Results section to make the derivation easier to follow and adapted Model 2 in the SI to include it as a tutorial example in that section.
- There is a new Table 1 with examples of linear independent *classes* of functions to clarify how the function decomposition is made.
- We have extended the discussion around Table 3 (former Table 2) to emphasize the benefits of our approach and how it contributes to a field where different methods provide inconsistent conclusions. We discuss the source of those discrepancies.
- We have added a brief discussion on the applicability and future extensions of our method to the so-called mixed-effects models. We had not considered this in the previous version but we think that our discussion opens new interesting problems to explore in that vast field.

We believe that the revised manuscript is stronger and more complete thanks to the issues raised by the referees. We hope it is deemed suitable for publication in PLoS Computational Biology.

Sincerely yours,

Mario Castro and Rob de Boer

Reviewer #1

The manuscript "Testing structural identifiability by a simple scaling method" focuses on an interesting problem regarding structural identifiability of mathematical models, or the ability to uniquely determine all or some of the parameters or parameter combinations based on observed data. The paper presents a concise, simple approach to this problem. It is an interesting article that could be of use in the field of mathematical modelling.

We thank the appreciation of the reviewer.

I was confused by Table 2, where the authors compared different methods for determining identifiability. Why do different methods arrive at disagreeing results for the same models? Could you explain and clarify how some tests find that the same model is globally structurally identifiable, while others find that the same model is unidentifiable (or locally structurally identifiable)?

We think that this is an important issue raised by the reviewer and we have added several paragraphs to address this point. Now, we discuss more thoroughly the source of the discrepancies that can be categorized into three:

- Local vs global structural identifiability (which is not an incompatibility as Global implies Local and our method is restricted to the latter);
- Conclusive vs not conclusive (which favors our method as it is not limited by any computational constraint). We also discuss the reasons why some methods do not provide a conclusive answer.
- Incompatible conclusions: Here, our method is compatible with the conclusions of differential algebra and hybrid methods such as Reaction network theory or STRIKE-GOLDD (that can be considered the state-of-the-art methods to test identifiability). Discrepancies with other methods are due to limitations or uncontrolled approximations when applied to complex problems.

Note: Former Table 2 is now Table 3.

I found the examples that were discussed and worked out in the supplementary information very useful, so I think that this information could be included in the main text.

Our aim with the SI was to create a comprehensive catalogue of cases to guide future readers to learn by example and leave the main text for the general procedure and comparison with other methods. However, we have adapted Model 2 in the SI to include it as a guided example in the main text to illustrate the main ideas.

Minor comments:

In the supplement in Section 2.2 in the first sentence, it says "when only x2 is observed". Should it say "when only x1 is observed"?

Typo right after (18) on p. 5 of supplement. It says, $u_x 1)1$ but should say $u_x 1 = 1$.

Typo right before section 2.9 on p. 10 of supplement. The u_d's should be u_δ's.

We have corrected these and other typos throughout the main text and the SI.

Reviewer #2

[...]This make this proposed work suitable for practitioners which have now generally a good background in statistics but lack a formation in algebra and are generally discouraged by the complexity of the theoretical ground needed to use the current identifiability checking methods.

We thank the reviewer his/her comments. We also appreciate all the suggestions about the structure of the manuscript. Our initial intention with the Supporting Information was to help the readers to learn by example but, clearly, some technical parts relegated to the SI must be in the main text to make it self-contained.

Major points: *The methods is interesting and indeed alleviate problems encountered by others and seems handy even for non specialist. However the writing does not enlighten that, right now it will be difficult for a biostatician to understand your method is relatively easy to use.*

1) You need to give a proper definition of functionally independence in the main paper. It is at the core of your work, it is the main reason why identifiability checking boils down to a simple set of equations. It is not a well known property among the biostaticians. Why not use the first equivalence characterization given in section 1.1 in Supporting information as the definition? It is the simpler formulation. You can say then that the original definition is more involved but let it in the supporting information. You need to say more about how we can construct these functions for some ODEs used in practice. Maybe insist on the case of ODEs with polynomial/rational vector fields? .

Thank you for this comment and constructive suggestions. In principle, we faced the dilemma of what to leave in the main text to make it more readable. To make the main text clearer, we have moved some definitions about functional linear independence and created the new Table 1 to show that the decomposition in Eq. (11) is not an obscure mathematical trick but simple inspection would do the job in most of the cases (with the help of the examples in the SI). We have also adapted Model 2 in the main text to illustrate the application of Box 1 to a nonlinear example.

2) The section "The main results" needs to be-rewritten. Its seems constituted of two blocks which hardly communicate. You have a first block describing how we end up with equations (16). Then you have a second block describing the derivation of the linear equation respected by the elasticity matrix components (just after equ 18). Both blocks are understandable but not their links. How you derive from the second block conditions under which F does not depends of other scaling factors is unclear. It will discourage readers.

We appreciate this comment as this is the central (technical) result in our work. Consequently, we have rewritten that whole section and have simplified the notation to help the reader to follow the derivation. In particular, we anticipate those two steps at the beginning of the section and reorganized the derivation to make it easier to follow.

3) Results of table 2 needs to be more clearly discussed. How do you interpret some methods conclude the model is identifiable and some others that it is not identifiable? Someone has to be wrong here, can you say which method it is? Be more precise about the category Not Conclusive Not Applicable. Does it means the ODE structure is not supported by the method? The algorithm/software crashed? The method does not converge after hours? etc

We agree that this Table is central and deserves more details (now this is Table 3). In the revised version we discuss more thoroughly the source of the discrepancies that can be categorized into three:

- Local vs global structural identifiability (which is not an incompatibility as Global implies Local and our method is restricted to the latter);
- Conclusive vs not conclusive,(which favors our method as it is not limited by any computational constraint) and;
- Incompatible conclusions: Here, our method is compatible with the conclusions of differential algebra and hybrid methods as Reaction network theory or STRIKE-GOLDD (that can be considered the state-of-the-art methods to test identifiability), and discrepancies with other methods are due to limitations or uncontrolled approximations when applied to complex problems.

We also devote some lines to clarify the *Not Conclusive/Not Applicable* column, where we summarize the main causes to fall into that category.

Minor points: Abstract

1) Maybe state more clearly from the beginning you are in a partially observed framework. That is appealing to practitioners and your methods handle it well.

We have partially rewritten the abstract to emphasize this point.

2) "The very structure of the model limits the ability to infer numerical values for the parameters, a concept referred to as structural identifiability:" A bit vague, the structural identifiability issue refers to different parameter values leading to the same model response making them unidentifiable from a noiseless and continuous system observation.

We have rewritten that sentence and replace it by a more actionable definition of structural identifiability:

A model is said to be structurally unidentifiable, if different quantitative sets of parameters provide the same observable outcome.

Minor points: Introduction

3) "Structural identifiability is a necessary condition for model fitting": To reformulate. Unfortunately, you will have a good model fitting with identifiability issues but the estimated parameter values will be nearly meaningless.

We have rewritten that paragraph and added a few statements to clarify this point:

Importantly, the quality of the fit does not guarantee that the estimated parameters are meaningful. In practice, this is both uncontrolled and misleading, as many fitting tools provide information about the goodness of fit but do not check sensitivity or identifiability.

4) "means that parameters can be estimated only in a limited subset of the space of parameters, i.e., only combinations of parameters are identifiable": can you explain the link?

We agree that this sentence was a bit obscure at this point in the Introduction and we have removed it. The discussion about identifiability groups is addressed elsewhere in the article.

Method

5) Page 3 line 69: "identifiabile"

6) Page 5: Equation 14 and 15, extra comma to remove

7) Page 5: line 119 I think it is j_1, j_2.

8) Page 5: In equation 17: inversion of differentiation between u_{λ_k} *and* $u_{\lambda_{j_1}}$.

Supporting information:

9) Page 5 typo: as x_1 *is observed (so,* u_{x_1})1).

10) Page 6: can you precise which are the linear independent functions you use for model 4?

Thank you for bringing up these typos. We have corrected these and all scattered in the text.

Reviewer #3

The method is based on mathematical results presented in the paper and is shown to perform well compared to other currently available methods, by concluding on the local identifiability of a number of models (detailed in the Supporting Information). The application of the method on a catalogue of models in the SI is very useful for the reader to understand how to apply the method in practice.

We are glad that the referee has enjoyed our contribution. We also thank him/her for the deep analysis and the interesting comments in the report which, no doubt, have helped to substantiate our main message and to improve the manuscript.

As mentioned in the last phrase of the discussion, my main concern is the lack of implementation of the method in a computational package. Even though the method is simple enough to be applied by making the computation by hand, the aim of making identifiability testing broadly accessible and more systematically done will be more easily reached if a computational method is also provided - especially for large dimension models.

We understand this concern raised by the reviewer and, definitely, we see the value of having the support of a computational tool. However, we still feel that it is out of the scope of the present method. In our experience, many modelers have a strong statistics and computational background but they often lack advanced analytical skills. Our method allows checking identifiability without those skills in many situations similar to those described in the Supporting Information. On the other hand, besides being beyond our expertise, designing a universal tool that can take a system of equations as input and perform this analysis is far from being straightforward and requires a deep software engineering background.

Other main points:

1) line 18: the notion of sensitivity is broadly used in the field, however the notion of elasticity is less common and could be defined here

We have included a proper definition of elasticity and its connection with the traditional sensitivity [new equations (3) and (4)].

2) line 78: "According to Sec. 1 in the Supporting Information...". Sec. 1 in the SI should be clarified to make sure that the reader can rely on it for the results presented in the main paper. In particular, it would be useful to have a clear theorem in the SI stating that any function can be split in a sum of independent summands. My understanding of the SI at the moment is only the fact that if some functions are independent, then any linear combination is equal to 0 only if the coefficients are equal to 0.

We have moved some material on functional linear independence from the SI to the main text to make it more self-contained. Regarding, the second comment, we do not conceive a general theorem to make that split but, rather, we provide (through the generalized Wronskian theorem) a way to test if different additive terms in an equation are linearly independent or not. In practice, most of the functions that are common in biophysical or pharmacokinetics models are already discussed in the examples of the SI. However, to make our method more straightforward, we have included the new Table 1 in the main text containing a short list of functions that are linearly independent to each other.

3) line 127: "Either all the coefficients $\beta_{kj} = 0$, meaning that Eq. (16) is not satisfied (we

will simply have $u_{\lambda_k} = 1$),". This phrase needs more explanation: in eq. (16), u_{λ_k} is written as a function F of $u_{x_{ml}}$ and $u_{\lambda_{jk}}$. If $\beta_{kj} = 0$, function F does not depend of the $u_{\lambda_{jk}}$, but what about the $u_{x_{m_l}}$ and how to conclude that $u_{\lambda_k} = 1$?

We appreciate this comment as this is the central (technical) result in our work. Consequently, we have rewritten that whole section and simplify a bit the notation to help the reader to follow the derivation.

5) It is common that the initial condition of the ODE corresponds to the equilibrium: if so, the initial conditions can sometimes be written as a function of the parameters of the ODE. How is this particular case handled by the method ?

This is also an interesting question and this sort of situation fits perfectly in our framework. Let us illustrate this with an example. Consider that one of those initial conditions can be written as

$$x_i(0) = \frac{p_1 p_2}{p_3}.$$

In this case, the scaled version would be

$$u_{x_i} x_i(0) = \frac{u_{p_1} u_{p_2}}{u_{p_3}} \frac{p_1 p_2}{p_3},$$

and we would have the additional identifiability equation

$$u_{x_i} = \frac{u_{p_1} u_{p_2}}{u_{p_3}}.$$

We have added this example to Model 3 in the Supporting Information.

6) It is also common to observe a combination of the x_i, for example $x_1 + x_2$ or $\frac{x_1}{x_1+x_2}$ and Model 11 in the SI only quickly mentions the case of the sum of the x_i. How can the method be adapted in these cases? As they are very common, it should be added in the article.

This is an interesting point. We have extended the dimensional argument at the end of Model 5 to discuss these situations and included explicitly the particular case of $\frac{x_1}{x_1+x_2}$ —new Eq. (28)— to clarify how our method also applies to generic functions of the observability conditions.

7) line 158 states that checking identifiability should be mandatory before fitting data. It should be added that it holds for the case of individual fits but is less clear in the case of population approaches based on linear mixed models, that are also broadly used (see for example Lavielle & Aarons, J. Pharmacokinet Pharmacodyn 2016, presenting PK models that are "non-identifiable at an individual level but can become identifiable at the population level if a number of specific assumptions on the probabilistic model hold").

This is another thoughtful question. This is indeed something that deserves more careful study (it is a field in itself!) but we have added a short paragraph in the discussion to show that our procedure is still valuable in this sort of situations:

For instance, if one considers the simple model

$$\dot{x} = (a+b)x,$$

a and b are not identifiable. However, if they are assumed to be drawn from, say, two exponential distributions with different means μ_a and μ_b, then the joint distribution for $\lambda \equiv a - b$

is given by

$$p(\lambda;\mu_a,\mu_b) = \frac{\mu_a\mu_b}{\mu_a-\mu_b}\left(e^{-\mu_b\lambda}-e^{-\mu_a\lambda}\right),$$

which is formed by two linearly independent functions (if $\mu_a \neq \mu_b$ *),* $e^{-\mu_b\lambda}$ *and* $e^{-\mu_a\lambda}$*, so* μ_a *and* μ_b *are identifiable as the unique solution of the identifiability equations*

$$\frac{u_{\mu_b}\mu_a u_{\mu_b}\mu_b}{u_{\mu_b}\mu_a - u_{\mu_b}\mu_b}e^{-u_{\mu_b}\mu_b\lambda} = \frac{\mu_a\mu_b}{\mu_a-\mu_b}e^{-\mu_b\lambda}$$

is $u_{\mu_b} = 1$ *(because of the exponential).*

Minor point and typos:

line 89-91: An explanation/proof that irreducible equations involving 2 or more parameters allow the identification of groups of variable that cannot be fitted independently could be added here or in the SI.

- *line 69: identifiable*
- *line 120: If we take*
- *SI - 1.2: eq(4)* $f_2(x_2)$ *and* $f_1(x_1)$
- *SI - 1.3: line before eq (8) "if the"*
- *SI - 2.2: line after eq (18)* $u_{x_1} = 1$
- *SI - 2.3: last paragraph "the fact that g in Eq (20a)"*
- *SI - 2.4: case 1,* $x_1(0) = K$
- *SI - 2.8: add a ")" at the end of the first line*

We have corrected these typos.
